# Gross Hematuria in Patients with Prostate Cancer: Etiology and Management

**DOI:** 10.1155/2013/685327

**Published:** 2013-03-24

**Authors:** Ofer N. Gofrit, Ran Katz, Amos Shapiro, Vladimir Yutkin, Galina Pizov, Kevin C. Zorn, Mordechai Duvdevani, Ezekiel H. Landau, Dov Pode

**Affiliations:** ^1^Department of Urology, Hadassah Hebrew University Hospital, P.O. Box 12000, Jerusalem 91120, Israel; ^2^Department of Pathology, Hadassah Hebrew University Hospital, P.O. Box 12000, Jerusalem 91120, Israel; ^3^Department of Urology, University of Montreal Hospital Center (CHUM), 1560 Sherbrooke E, Montreal, Quebec, Canada

## Abstract

The objective of the study is to assess the etiology and prognosis of gross hematuria (GH) in patients with carcinoma of the prostate (CAP). From 1991 to 2011, 81 men (mean age 74.3 years, SD 6.5) with CAP were hospitalized with GH. Primary treatment of CAP was radical surgery in 13 patients (group 1) and nonsurgical therapy in 68 (group 2), mostly radiotherapy (35 cases) and hormonal treatment (25 cases). The common etiologies of GH in group 1 were bladder cancer (38.5%) and urinary infection (23%). In contrast, CAP itself caused GH in 60% of the patients in group 2. Thirty-nine patients (48%) required transurethral surgery to manage GH which was effective in all cases; nevertheless, the prognosis of group 2 patients was dismal with median overall survival of 13 months after sustaining hematuria, compared to 50 months in group 1 (*P* = 0.0015). We conclude that the etiology of GH in patients with CAP varies according to primary treatment. After radical prostatectomy, it is habitually caused by bladder cancer or infection. When the primary treatment is not surgical, GH is most commonly due to CAP itself. Although surgical intervention is effective in alleviating hematuria of these patients, their prognosis is dismal.

## 1. Introduction

The frequency of GH in patients with CAP is unknown. GH can be a result of CAP itself, a side effect of previous treatments (acute or chronic toxicity of radiotherapy, stone formation on a suture after surgery) or unrelated to CAP. 

GH after external beam radiotherapy is not uncommon, but is usually mild and self-limited [1]. Its occurrence is dependent on bladder wall dose-volume [2], and it is more frequent in patients that had previous transurethral prostatectomy [3]. Macrohematuria after brachytherapy is rare (less than 1%) [4]. It seems that hormonal therapy is a protective factor for late hematuria after high-dose radiotherapy for CAP [5]. The effect of finasteride, known to prevent GH due to benign prostatic enlargement [6] in patients with malignant disease, is unknown. The frequency of GH after radical prostatectomy and cryotherapy and in patients receiving hormonal treatment is not documented in the literature.

The management of GH in patients with CAP can be quite difficult. Various treatments were suggested including finasteride, radiotherapy, antifibrinolytics, bladder irrigations with alum solution and transurethral surgery, and angioembolization, none with proven effectiveness [7]. Total pelvic exenteration was also suggested in desperate cases [8]. 

GH in patients with CAP is, therefore, a complex condition. Multiple factors should be taken into consideration when caring for a patient with GH and a history of CAP, in addition to the generally important parameters (age, smoking history, symptoms of infections, etc.). These factors include disease stage and grade, when was CAP diagnosed, and how was it treated? The relative importance of each of these factors to diagnosis and management of GH is unknown and was examined in this study. The effectiveness of various treatments (especially transurethral surgery) for curing GH and patients' prognosis after sustaining GH was also addressed.

## 2. Materials and Methods

We systematically surveyed a tertiary hospital database (1991–2011) for patients with known CAP who were hospitalized with GH. Parameters studied included patient's parameters (patients' age, age at diagnosis of CAP), tumour parameters (stage, grade, and time of diagnosis of CAP) treatment for CAP, etiology of GH, method of diagnosis done, GH treated, and prognosis after treatment. An institutional review board approved the study (Approvement no. 0070-11-HMO). 

All patients included in the study had at least grade 2 hematuria according to the Radiation Therapy Oncology Group (RTOG)/European Organization for the Research and Treatment of Cancer (EORTC) Common Toxicity Criteria, version 2.0. (a scale in which grade 1 is microhematuria, and grade 2 is macroscopic hematuria not requiring blood transfusion or surgical intervention, grades 3 and 4 are severe hematuria requiring accordingly minor or major surgery) [9].

Statistical Analysis was done using the JMP software (Cary, NC, USA). The Student's *t*-test was used to compare continuous variables while Fisher's exact test and chi square test were used to compare categorical variables. Kaplan-Meier method with the log-rank test were used to evaluate survival. A *P* value <0.05 was considered significant. 

## 3. Results

During the study period, 81 men (mean age 74.3 years, SD 6.5 years) with prostate cancer were admitted to hospital with GH. Patients' and tumour characteristics are presented in Table 1. Median patients' age upon diagnosis of CAP was 73 years, median PSA upon diagnosis was 14.3 ng/mL, and median Gleason score was 7. Primary treatment for prostate cancer was radical surgery in 13 patients and nonsurgical in 68 (radiotherapy in 35 cases, hormonal treatment 25, cryotherapy and surveillance each in 3, and transurethral prostatectomy in 2 patients). As expected, patients treated by surgery were significantly younger and had a lower PSA. Pathological analysis of the surgical specimens of patients that underwent radical prostatectomy showed Gleason scores of 5-6, 7, and 8–10 in 7, 5, and one patients, respectively, and pathological stages were T2 and T3 in 7 and 6 patients, respectively. In 5 patients (38%), surgical margins were positive for cancer. Mean patients' age at onset of GH was 76.5 years (SD 7.8 years). The time from diagnosis of prostate cancer to onset of GH was 50 months (SD 50 months) in nonoperated patients and 67 months (SD 59 months) in operated patients (*P* = 0.25).

Diagnosis of hematuria was accomplished by cystoscopy in 66 patients (81.5%) and by urine culture in 12 patients (15%). The etiologies of GH according to primary treatment of CAP are also presented in Table 1. As can be noticed, the etiologies of GH varied according to the primary treatment of prostate cancer. The common etiologies of GH in operated patients were bladder cancer (38.5%) and infection (23%). In contrast, prostatic bleeding (mostly due to malignant disease) caused 65% of the GH in nonoperated patients. 

**Figure 1:**
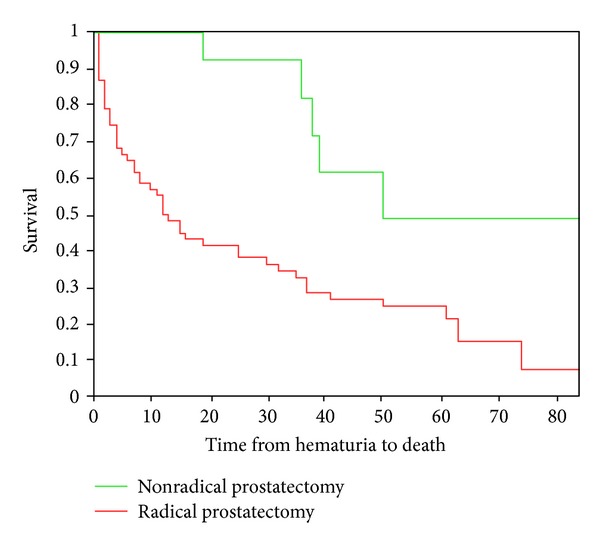
Overall survival of patients with prostate cancer after sustaining gross hematuria according to initial treatment of prostate cancer (*P* = 0.0015).

The treatments of GH and patients' prognosis are presented in Table 2. About half of all patients required surgical intervention (GH grades 3 and 4). Surgery was very effective and cured GH in all patients. Patient prognosis after sustaining GH was dependent on the initial treatment for CAP (Figure 1). Median overall survival after radical prostatectomy was 50 months and median disease-specific survival not reached. On the contrary, median overall survival was only 13 months in patients that did not had radical surgery and median disease-specific survival 19 months (*P* = 0.0015).

## 4. Discussion

In this research, we evaluated all patients with CAP presenting to a tertiary hospital with GH within 20 years. It was found that etiology of GH and patients' prognosis could be deduced from the initial treatment of CAP (Table 1). While bladder cancer and infection were the common etiologies of GH in operated patients, CAP itself caused 60% of all GH in nonoperated patients. CAP did not cause GH in any of the patients that had radical prostatectomy, including cases with positive surgical margins. Avoidance of this severe and devastating symptom may be considered a noteworthy benefit of radical surgery treatment over nonsurgical therapies of CAP. 

Diagnosing the etiology of GH was not difficult in most cases. Urine culture and cystoscopy provided the diagnosis in 95% of the cases. These two tests should be considered mandatory in any patient with GH.

Various treatments for GH in CAP patients were proposed in the literature including hormonal manipulation, antifibrinolytics, embolization of the internal iliac arteries, and intravesical instillations of various agents [7]. Palliative radiotherapy for GH was reported to alleviate hematuria in 81% of the patients, 6 weeks after completion of treatment. But response rate dropped to 29% after 7 months [10]. Radiotherapy is evidently not an option in patients that were previously irradiated. Considering the effectiveness of transurethral surgery in curing GH in patients with CAP (100% cure rate), this should be the primary therapeutic mode in most cases of severe GH.

The prognosis of patients with CAP that developed GH was dependent on initial therapy for CAP (Table 2 and Figure 1). Patients that did not have radical prostatectomy had a poor prognosis with median overall survival of 13 months, as opposed to 50 months in patients that had surgery (*P* = 0.0015). This stems from both a more advanced CAP at diagnosis of cancer in the “nonoperated patients” (PSA of 7.4 ng/mL in operated patients compared to 18.5 in nonoperated patents) and from the more advanced stage during hematuria. 

## 5. Conclusions

The etiology of GH in patients with CAP varies according to primary treatment for CAP. After radical prostatectomy, it is habitually caused by bladder cancer or infection and is almost never related to prostate cancer. On the contrary, when the primary treatment is not radical surgery GH is most commonly due to CAP itself. Diagnosis of GH can be accomplished in most cases by cystoscopy and urine culture. The management of these patients is difficult; transurethral surgical intervention is often needed. Surgery is very effective in alleviating GH but the patients' prognosis is dismal. 

## Figures and Tables

**Table 1:** Characteristics, grade, and etiology of gross hematuria in patients with prostate cancer according to cancer primary treatment.

Primary treatment	Radical prostatectomy	Other treatments	*P* value
Number of patients	13	68	
Mean age (SD)	65.5 (8.46)	74.3 (7.1)	0.003
Median PSA (ng/mL)	7.4	18.5	0.02
Median Gleason score	7	7	
Hematuria			
Grade II	5 (38.5%)	37 (54.4%)	
Grades III*∖*IV	8 (61.5%)	31 (45.6%)	0.369
Etiology of hematuria			
Prostatic bleeding	—	44 (64.7%)	
Prostate cancer	—	41 (60.3%)	
Benign disease	—	3 (5.4%)	
Bladder cancer	5 (38.5%)	4 (5.9%)	
Infection	3 (23.1%)	9 (13.2%)	
Urolithiasis	1 (7.7%)	1 (1.5%)	
Suture	2 (15.4%)	—	
Radiation cystitis	—	9 (13.2%)	
No diagnosis	1 (7.7%)	—	
DIC	1 (7.7%)	1 (1.5%)	

**Table 2:** Treatment and prognosis of patients with prostate cancer and gross hematuria according to cancer primary treatment.

Primary treatment	Radical prostatectomy	Other treatments	*P* value
Treatment of hematuria			
Transurethral resection of prostate	—	26 (38.2%)	
Conservative management	1 (7.7%)	27 (39.7%)	
Transurethral resection of bladder cancer	5 (38.5%)	4 (5.9%)	
Suture removal	2 (15.4%)	—	
Antibiotics	3 (23%)	9 (13.2%)	
Stone removal	1 (7.7%)	1 (1.5%)	
Heparin	1 (7.7%)	1 (1.5%)	
Median overall survival (months)	50	13	0.0015
Median disease-specific survival	Not reached	19	0.0035
